# Learning in Neurofeedback is Heterogenous and Does Not Guarantee ADHD Symptom Improvement

**DOI:** 10.1007/s10484-026-09774-w

**Published:** 2026-02-27

**Authors:** Elizaveta Kuznetsova, Roger de Beus, L. Eugene Arnold, Evgenii Rudakov, Benjamin Ultan Cowley

**Affiliations:** 1 Faculty of Educational Sciences, University of Helsinki, Siltavuorenpenger 5A, 00170 Helsinki, Finland; 2 Department of Psychology, University of North Carolina Asheville, Asheville, NC USA; 3 Department of Psychiatry and Behavioral Health, Nisonger Center, College of Medicine, Ohio State University Wexner Medical Center, Columbus, OH USA; 4 Cognitive Science, Department of Digital Humanities, Faculty of Arts, University of Helsinki, Helsinki, Finland

## Abstract

Neurofeedback (NFB) is a non-invasive intervention for ADHD that aims to train self-regulation of brain activity. Despite decades of clinical application, evidence for its efficacy remains mixed, and the mechanisms—particularly the role of NFB learning in ADHD symptom reduction—are still not well understood. This study advances the NFB field by introducing both theoretically grounded and data-driven frameworks that conceptualize learning as a dynamic, individualized process. Data were drawn from a 25-month longitudinal randomized controlled trial of theta/beta ratio (TBR) NFB treatment in children with ADHD (*N* = 100; ages 7–10). Moving beyond prior work that characterized learning through binary classifications and group-level averages, we captured within- and between-session training dynamics and applied clustering methods to define distinct learner profiles, highlighting the heterogeneous nature of NFB responses. Our results demonstrate that while most participants learned to regulate TBR effectively, individual learning did not reliably predict symptom improvement. Instead, linear mixed-effects model results indicated that non-specific factors such as participant engagement and training context were more predictive of clinical outcomes. Moreover, variability in learning curve shapes—typically overlooked in standard NFB studies—suggests that NFB learning does not follow a uniform pattern, with individuals engaging through distinct mechanisms aligned with either operant-conditioning or skill-acquisition models. These insights advance theoretical understanding of NFB mechanisms and call for individualized, mechanism-informed approaches to optimize treatment outcomes and deepen clinical interpretation.

## Introduction

Neurofeedback (NFB) is a non-invasive treatment with minimal side-effects, in which patients are presented with information about their brain electrical activity to enable conscious control of their brain waves. NFB has been proposed as a complementary option for treatment of ADHD and has a long history of clinical application (Arns et al., 2014; Lubar & Shouse, 1976; Shouse & Lubar, 1979). However, 50 years later debate continues over the specific factors behind the improvements in many outcome measures, including parent-rated symptoms in this intervention. Unblinded randomized controlled trials (RCTs) generally show a medium benefit over the control condition (Cortese et al., 2016; Peterson et al., 2024) while blinded RCTs generally show similar improvement in both treatment groups, a non-specific effect (e.g. Neurofeedback Collaborative Group, 2021). Thus, existing evidence is mixed and often non-specific, and the mechanisms of NFB are still poorly understood. The critical question remains: *What characterizes those individuals who gain true therapeutic benefit from NFB?*

In this study, we directly address the disconnect between EEG-derived learning metrics and clinically meaningful outcomes—a gap that continues to complicate both the interpretation of findings and their application in clinical practice.

A particularly important question is how the learning in NFB contributes to its efficacy (Zuberer et al., 2015). A recent systematic review on learning in NFB (Kuznetsova et al., 2023) found that very few clinical trial reports have dealt with the heterogeneity of NFB learning, nor analyzed whether NFB clinical efficacy is dependent on NFB learning. Most of the surveyed research papers presented data on the group level and reported pre-treatment to post-treatment differences. However, it is crucial to distinguish individual patients, because pooling data of ADHD patients who might benefit differently from NFB treatment not only diminishes individual effect sizes (cf. Cortese et al., 2016; Micoulaud-Franchi et al., 2014; Sonuga-Barke et al., 2013) but also hides any qualitatively different effects manifested in different subgroups (learners vs. non-learners) (Kuznetsova et al., 2023). As a result, the lack of noticeable group-level differences in self-regulation improvement from pre- to post-treatment may mask significant individual improvements that would help elucidate the true mechanism of NFB effect.

The lack of reporting on learning characteristics in the existing literature may stem from the challenge of defining learning within the context of NFB. While most behavioral tasks typically have clear performance metrics, assessing performance in NFB is more complex due to unstandardized protocols, the role of the treatment provider, covariate trained metrics (e.g. myogenic activity), and uncertainty regarding what constitutes ‘good’ performance. This heterogeneity persists even within so-called “standard” protocols: for instance, a systematic review of slow cortical potential (SCP) NFB documents substantial variation across studies in the number and length of trials, the balance of feedback versus transfer trials, reinforcement/token systems, artifact handling, and transfer practices (Hasslinger et al., 2022). As a result, studies that report learning parameters often use differing definitions of learning, contributing to the lack of consensus on what successful learning entails (Arns et al., 2020; Hasslinger et al., 2022; Kuznetsova et al., 2023). This highlights the urgent need to better understand learning in NFB by developing standardized, theory-driven operational definitions and robust analytical frameworks that can account for both individual variability and the complexity of the learning process.

In its early days, NFB was viewed as a form of operant conditioning where subjects learned to automatically regulate EEG traits through external reinforcement. At that time, ADHD was believed to stem from irregularities in EEG-recorded brain activity, and treatment approaches were aimed at correcting these neurophysiological disruptions (Gevensleben et al., 2009; Lubar & Shouse, 1976; Shouse & Lubar, 1979). Later on, Gevensleben and colleagues (2014) proposed the skill-acquisition model, wherein conscious effort and skill are required to change the EEG state. Thus, learning in the skill-acquisition model would be viewed as progressive internal development of expertise, characterized by enhanced volitional control over EEG states (Sherlin et al., 2011). This in fact reflected the widely-held clinical view that subjects must self-discover some strategy, which was idiosyncratic and thus unteachable, to properly benefit from NFB. Strehl (2014) attempted to reconcile the hypotheses of operant conditioning and skill acquisition, drawing a parallel to Fitts’ theory of motor learning (Fitts & Posner, 1967), which suggests that cognitive learning is a prerequisite for the automation of skills.

In this study, for the first time, we systematically characterize and parametrize patient learning processes during and after NF training. We analyze data from the International Collaborative ADHD Neurofeedback (ICAN) RCT conducted over five years by the Neurofeedback Collaborative Group (Arnold et al., 2021), in which each participant engaged for approximately 25 months overall. To examine the nature of NFB learning, and how its distinct trajectories and modulators contribute to treatment outcomes, we explore the following key questions:

- RQ1. How does NFB learning and non-specific factors relate to symptom outcome?

While NFB aims to facilitate self-regulation of brain activity, it remains uncertain whether improvements in clinical symptoms are driven by successful engagement with the learning process, or by non-specific factors such as training engagement, motivation, and amount of sleep. In other words, do patients benefit from *what they learn (*Arns et al., 2014; Holtmann et al., 2014), or from *the experience of training itself (*Bakhshayesh et al., 2011; Veilahti et al., 2021)?

Previous work has highlighted the importance of achieving specific neurophysiological targets, such as modulation of theta and beta rhythms, in producing behavioral change (Gevensleben et al., 2012). However, few studies have explicitly linked these objective markers of learning to treatment outcomes (e.g., Drechsler et al., 2007; Gevensleben et al., 2012, p. 20; Leins et al., 2007). Those who did mostly presented gathered data on the group level and reported only pre- to post-treatment differences. Only three studies—Baumeister et al. (2018) and Janssen et al. (2017) in children, and Veilahti et al. (2021) in adults— have explored individual learning trajectories; and still there, individual learning was measured simply as a linear slope across sessions, which may oversimplify the complexity of NFB learning.

At the same time, a growing body of research suggests that non-specific factors—such as motivation, comorbid conditions, and treatment setting—can significantly moderate treatment response (Bakhshayesh et al., 2011; Roley-Roberts et al., 2023). For instance, Roley-Roberts et al. (2023) found that in the ICAN RCT, anxiety comorbidity predicted poorer outcomes at post-treatment, while oppositional-defiant comorbidity predicted better long-term outcomes.

In this study, we take a combined approach: we examine how both neurophysiological learning indicators and non-specific factors (e.g., medication status, age of participants at intake, sleep quality, training setting) relate to clinical change. By modeling the granular *components* of learning using three operational metrics—within-session gains, between-session improvement, and performance consistency—we aim to determine which aspects of learning, if any, are predictive of treatment success, and how these interact with broader contextual and individual factors.

- RQ2. How is learning modulated by non-specific effects (e.g. sleep, medication status, engagement levels during NFB sessions, etc.)?

The idea that NFB learning might be a form of skill acquisition implies that motivational, attributional, and personality factors might play a stronger role (Gevensleben et al., 2014). For instance, participants’ beliefs about their ability to control technological devices, or their use (or lack) of effective mental strategies during training, can influence outcomes (Kober et al., 2013). Studies of children with ADHD have commonly observed self-perception biases; many tend to overestimate their own abilities, including self-efficacy and performance (Owens et al., 2007), while paradoxically, lower self-esteem has been associated with slightly better learning outcomes (Newark & Stieglitz, 2010).

More recently, Veilahti et al. (2021) provided empirical evidence that personality and psychopathology traits measured at baseline can predict NFB learning. Specifically, higher scores in generalized anxiety (GAD) and behavioral inhibition system (BIS) were associated with greater learning success, whereas high dissociation system scores (DES) predicted poorer outcomes. These findings reinforce the idea that the NFB learning process is embedded within a broader psychological and contextual framework. Identifying such modulators is critical for understanding individual variability in learning trajectories and for optimizing the conditions under which NFB is delivered (Feo et al., 2025).

- RQ3. Does neurofeedback performance approximate any distinct learning curve (e.g., exponential, logarithmic, power-law, linear), and do the observed curves reflect different mechanisms of learning?

Building upon an already established framework of learning in stages (Fitts & Posner, 1967; Karni & Sagi, 1993; Kuznetsova et al., 2025), we propose that learning is likely non-uniform and unfolds through distinct stages of acquisition, consolidation, and maintenance. Learning curve analysis can help reveal these distinct learning phases and help clinicians design tailored interventions appropriate for each learning stage, optimizing NFB session duration, scheduling, and intensity. The shape of the learning curve might also provide insight into the underlying cognitive and neurophysiological processes. Linear curves typically suggest operant conditioning, characterized by repetition and reinforcement (Staddon & Cerutti, 2003), while power-law or logarithmic curves might better capture cognitive skill acquisition dynamics and early unmasking of latent capacity before transitioning into more gradual learning-driven improvements (Fitts & Posner, 1967; Newell & Rosenbloom, 1982; Van Der Veer et al., 2020).

To address our three research questions, our study proceeds along two complementary tracks. First, we apply a theory-driven framework that operationalizes learning based on established models and expected NFB outcomes. Using this framework, we propose eight distinct learner profiles, each characterized by unique temporal features and performance traits.

Second, we pursue a data-driven approach, leveraging unsupervised clustering to uncover emergent patterns in EEG self-regulation. Together, these dual tracks based on within- and between-session training metrics allow us to evaluate NFB learning both through the lens of theoretical expectations and through patterns arising directly from the data, offering a richer and more individualized understanding of the mechanisms and moderators underlying NFB treatment efficacy.

## Methods

### Participants

The data was collected from 142 ADHD child patients ages 7–10: 84 subjects in training group (Active), who received NFB based on their real-time EEG recordings, and 58 subjects in sham group (Control), who received NFB based on pre-recorded, age-matched NFB sessions’ EEG signal. The analysis focused on participants who completed all 38 sessions and had sufficient data available, resulting in a final sample of 100 participants (Active = 58, Control = 42).

### Inclusion Criteria

Inclusion criteria were as follows: age between 7 and 10 years (inclusive); IQ of 80 or higher; a rigorous DSM-5 diagnosis of ADHD (either inattentive or combined presentation), confirmed through a structured interview conducted by a doctoral-level clinician, and supported by parent and teacher ratings at least 1.5 standard deviations above the norm for DSM-5 ADHD symptoms, as assessed using the Conners Parent and Teacher Rating Scales (Conners, 2008); and an eyes-open theta/beta power ratio of ≥ 4.5 at Cz or Fz, as measured by the Lubar Monastra Assessment Suite (Thought Technology, Toronto, ON, Canada).

### Exclusion Criteria

Participants were excluded if they had a comorbid disorder requiring psychoactive medication other than a psychostimulant, or a medical condition necessitating chronic systemic medication with potential psychoactive effects. Other exclusions included more than five prior NFB sessions, concurrent psychosocial treatment, sleep-related disorders, convergence insufficiency, planned school transfers, or upcoming ADHD treatment changes. Vitamin D deficiency/insufficiency was a temporary exclusion criterion until replenished. Psychiatric medications had to be discontinued for a specified period before baseline, and stimulant users withheld medication for five days—at least three of which were school days—prior to major assessments. Ambiguous cases were reviewed by a cross-site panel.

### Design

The design was a 2-site, parallel-group, double-blind randomized comparison of active NF to “sham” NF (Control) for up to 38 treatments in a 14-week period, with 6-, 13-, and 25-month follow-up (dated from baseline). At each of 2 location sites – Ohio State University (OSU) in Columbus, OH and Neurofeedback Clinic in Asheville, NC – written parental consent and child assent were obtained with procedures and documents approved by the university Institutional Review Board at OSU. Participants were recruited by university press release, by notification of university and community child psychiatrists, pediatricians, and other professionals, and by advertisement. Eligible children were randomized to Active NF versus Control in a 3:2 ratio, stratified by site and current ADHD medication. Randomization was supervised by the study biostatistician and implemented remotely in the software by an unblinded co-investigator who had no contact with participants. The follow-ups were considered an integral part of the RCTs because enduring benefit was important to justify the expense and effort of labor-intensive NFB.

### Measures

The standardized diagnostic interview at screening and follow-ups was the Children’s Interview for Psychiatric Syndromes Child (ChIPS) and Parent (P-ChIPS) versions. Repeated outcome measures (unmedicated for 5 days before the assessments) included the following: combined parent & teacher ratings on the Conners DSM-5 ADHD Inattentive Scale, other psychiatric symptoms, functional impairment, sleep, neuropsychological tests, assessments of EEG and Event Related Potentials (ERP), monitoring of adverse effects and changes of concomitant treatments, treatment satisfaction, blinding, and compliance/adherence. Neurofeedback Collaborative Group recruited from August through February so that the child could have the same teacher for baseline, mid-treatment, and end-of-treatment assessments during the 3-month treatment.

### NFB Training

NFB training used the FDA-registered EEGer system (EEG Software LLC, Northridge, CA) with either a Thought Technology or BrainMaster Atlantis amplifier. Amplifier assignment was randomized and balanced by the treatment group. The head site with the more deviant TBR (Fz or Cz) was selected for training, as measured during screening. The right ear was used as the reference and the left ear as the ground.

In the NFB training program, children interacted with computer games that provided real-time feedback contingent on EEG activity. Each session used a game-based interface: at the start of each session, the child selected one game from a set of 10 available options, and this game provided the feedback display for the session. The goal was to help participants learn to regulate their attention by decreasing theta activity, increasing beta brainwave activity, and minimizing muscle tension (EMG). Feedback was delivered visually and aurally through changes in game elements—such as moving characters, earning points, or triggering animations—when the children met specific brainwave thresholds.

Children could earn up to 20 points per session based on improvement relative to the previous session; accumulated points were exchanged for a monetary reward to support engagement. Trainers played an active and supportive role—guiding children in understanding their goals, identifying effective strategies, and maintaining focus and motivation of the participants. Coaching targeted behavioral correlates of the trained state—particularly minimizing muscle artifact—by helping children experiment with posture, paced breathing, and reducing jaw/face tension, and by prompting active engagement (‘looking with a purpose’) via brief, concrete goals (e.g., on-screen targets or time-based challenges such as maintaining threshold success for a set duration).

Each treatment session was organized into five consecutive training blocks (hereafter “tasks”), separated by brief pauses. During each of the five tasks, we recorded the following neural parameters:

1. **Score**: The number of half-second epochs in which participants met the reward criteria (theta µV below the threshold, beta µV above the threshold, EMG µV below the threshold, and Trace A µV below the threshold).
2. **Theta**: The average oscillatory power within the 4 to 8 Hz frequency band.
3. **Beta**: The average oscillatory power within the 13 to 21 Hz frequency band.
4. **TBR**: The average ratio of theta to beta power.

After five sessions, the thresholds for theta and beta were averaged and re-adjusted, remaining constant for the rest of the sessions. We also recorded the following artifacts:

- **Trace A**: The overall EEG amplitude, which captured eye blinks and significant EEG elevation due to gross movement (1–60 Hz). This had to meet a set threshold before any other metrics could be rewarded.
- **EMG**: Muscle artifact in the 45–60 Hz range.

Both groups received real-time feedback on artifact parameters, which remained consistent throughout the remainder of the training sessions.

Each training session consisted of five tasks, with a break between each. The breaks between tasks lasted several seconds to minutes and were used to record data and provide verbal and visual feedback/coaching to the children regarding their previous task’s performance. The duration of tasks increased across blocks as follows:

- Block 1 (Sessions 1–5): Five 5-minute tasks.
- Block 2 (Sessions 6–10): Five 6-minute tasks.
- Block 3 (Sessions 11–19): Five 7-minute tasks.
- Block 4 (Sessions 20–29): Five 8-minute tasks.
- Block 5 (Sessions 30–38): Five 9-minute tasks.

### Primary Outcome

The primary outcome was the DSM-5 ADHD Inattentive scale on the Conners 3 combined parent and teacher ratings (Conners, 2008). This rates symptoms/problems from 0 (no problem) to 3 (severe) and yields 12 sub-scales including DSM-5 inattention symptoms. It has norms and T-scores. Teacher- and parent-rated outcomes were collected repeatedly (baseline, mid-treatment, post-treatment, and three follow-up time points; additionally, for unmedicated participants, ratings were also collected after 10 and 30 sessions). For the primary outcome, we created a single “combined informant” score at each measurement point by taking the arithmetic mean of the available parent and teacher total scores.

### Pre-processing

EEG data was collected from the NF software delivering the intervention (EEGer). Data was sampled at 256 Hz per second, then averaged by task in each session. If Trace A was above threshold, all other values became zero until the trace was back in range. All of these types of zeroes were removed from the data before averaging. To manage outliers, we excluded the lowest and highest 0.05% of TBR, theta, beta and EMG amplitude values.

Symptom data were imputed when missing: if follow-up values were absent, we carried forward the last available measurement. For participants on medication (~ one third of the sample), medication was *not* paused at sessions 10 and 30. Consequently, symptom ratings at these sessions are not comparable to the off-medication assessments at baseline, sessions 20 and 40, and follow-ups. We therefore coded sessions 10 and 30 as missing for medicated participants; data from unmedicated participants were retained.

Additionally, because each participant had two threshold settings—an initial set applied to sessions 1–5 and a recalibrated set (theta and beta averaged over sessions 1–5) applied to all subsequent sessions—we excluded sessions 1–5 from the percent-time-in-reward analyses to avoid calibration-induced bias.

### Modelling Methods

The entire conceptual flow of the study hinged on the question of how we parameterize the performance data. In this study we aligned existing theoretical ideas of how learning in NFB works with operational concepts. At its core, NFB learning data can be viewed as time-series data reflecting changes in EEG traits over time. Such data inherently capture dynamics at multiple temporal scales—both within individual sessions and across multiple training sessions. Within-session analysis examines immediate responsiveness and consistency, while between-session analyses track sustained changes in EEG patterns. We thus developed three operational concepts (metrics) to describe within- and between-session learning:

- **Gain**: signed magnitude of change from the beginning of study training (Task 1 Session 1) to the end (Task 5 Session 38).
- **Focus**: signed change of variance from the beginning of study training (Session 1) to the end (Session 38),
- **Consistency**: signed magnitude of within-session changes on average.

To quantify *gain*, we constructed a linear model for each participant, using Task as the independent variable (IV) and training parameters (e.g. TBR, theta amplitude, beta amplitude, Score), as dependent variables (DVs). We then extracted the difference between the ending point and the intercept, representing the magnitude of change over the course of training.

To quantify *focus* we calculated the standard deviation within the Session and constructed a linear model for each participant, using Session as IV and training parameters as DVs. We then extracted the difference between the ending point and the intercept, representing the change of variance from beginning to the end of training. This metric reflects the change in the variability of performance across the training.

To quantify *consistency*, we built a linear model for each session and each participant, using Task in session as IV and training parameters as DVs. We then extracted the slope from each session’s model and averaged these values to determine the overall direction and size of within-session changes for each participant. This metric serves as a measure of responsiveness to the training on a within-session level which, indicating the ability to obtain gain within sessions, even if performance returns to baseline between sessions.

We employed two complementary approaches to analyze our data. Our *theory-driven approach* is grounded in established models of learning and expected NFB outcomes. This approach assumed that effective NFB would lead to specific changes in brain activity—namely, a decrease in TBR and theta amplitude, an increase in Beta amplitude, and an overall improvement in training performance. Guided by these expectations, we defined eight distinct learner profiles (see below) and assigned participants to these profiles based on their observed performance patterns.

However, this theory-driven perspective assumes the training protocol operates exactly as intended. Thus, we also adopt a *data-driven approach* to account for the possibility that the multiple training parameters may interact in unforeseen ways. Here we no longer impose predefined expectations of who the “perfect learners” should be. Instead, we let the patterns of performance reveal themselves, allowing for a more flexible and empirically grounded classification.

### Theory-driven Approach

Our analysis focuses on TBR performance since TBR is the phenomenon of highest clinical relevance. 

We clustered participants into “high” vs “low” responders for each operational concept (gain, focus, and consistency) within each training parameter (TBR, theta, beta, and Score). To do so, we applied the *k*-means clustering approach (k=2) with 2000 bootstrap iterations, yielding a distribution of midpoints between the two cluster centers . Participants whose values fell between the 25th percentile (lower bound) and 75th percentile (upper bound) of the midpoint distribution were excluded from group assignment, ensuring only those with clearly distinct learning patterns were classified.

**Fig. 1:**
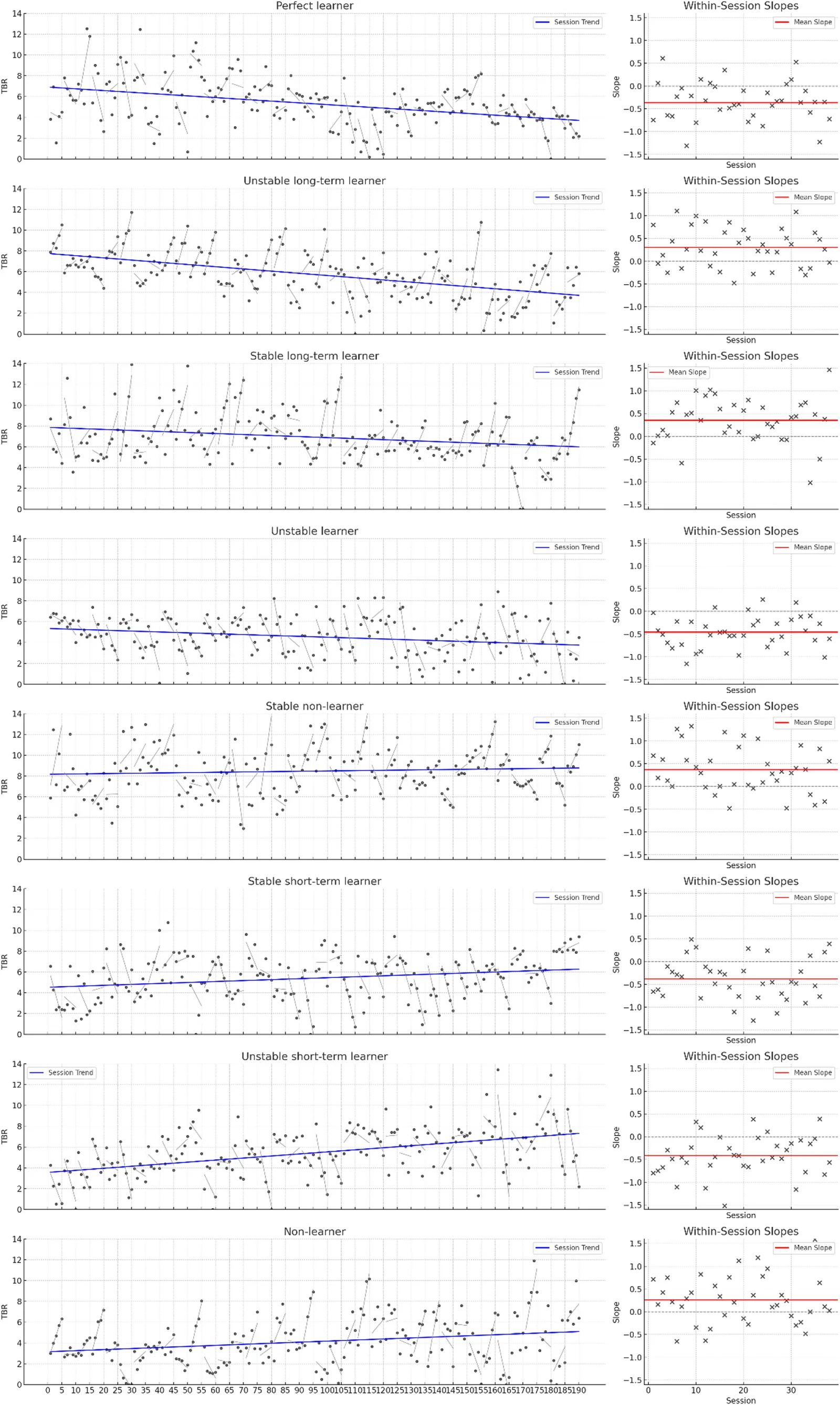
*Simulated TBR performance trajectories and within-session learning dynamics across eight learner profiles.* Left panels show task-by-task TBR values across 38 sessions. Blue lines represent overall session trends (*gain*), while gray lines indicate within-session linear slopes (*consistency*). Right panels display the slope values for each session, with red lines marking the average slope (*consistency*). Stable profiles exhibit decreasing within-session variability (*focus*), while unstable profiles show increasing dispersion over time

Given two levels of three learning metrics, we characterize eight (2^3^ = 8) distinct learner profiles, defining participant success for each training parameter (TBR, etc.). Figure 1 illustrates the simulated TBR performance trajectories and within-session learning dynamics across eight distinct learner profiles:

1. *Perfect learner.* This participant improves all three operational concepts of training parameters. Perfect learner successfully regulates both within and across sessions in a stable and consistent manner. Perfect learner represents the ideal NFB outcome, successfully applying learned strategies.
2. *Unstable long-term learner.* This participant improves the gain, but their focus deteriorates and mean within-session gain does not represent the short-term desired responsiveness to treatment. Unstable long-term learner shows long-term improvement but lacks short-term control.
3. *Stable long-term learner* improves across sessions and becomes more consistent, while does not demonstrate significant within-session learning.
4. *Unstable learner* demonstrates learning both within and between sessions but in an inconsistent manner.
5. *Stable non-learner* maintains a consistent performance pattern without between- and within-session improvement.
6. *Stable short-term learner* improves within sessions but fails to carry it over to future sessions. Performance stabilizes within a session, suggesting local learning effects.
7. *Unstable short-term learner.* Shows within-session improvement in some sessions, but not others, in an erratic manner. Unlike *stable short-term learners*, their improvements are inconsistent across trials.
8. *Non-learner.* These participants do not exhibit learning in the NFB task on any of the metrics.

**Fig. 2:**
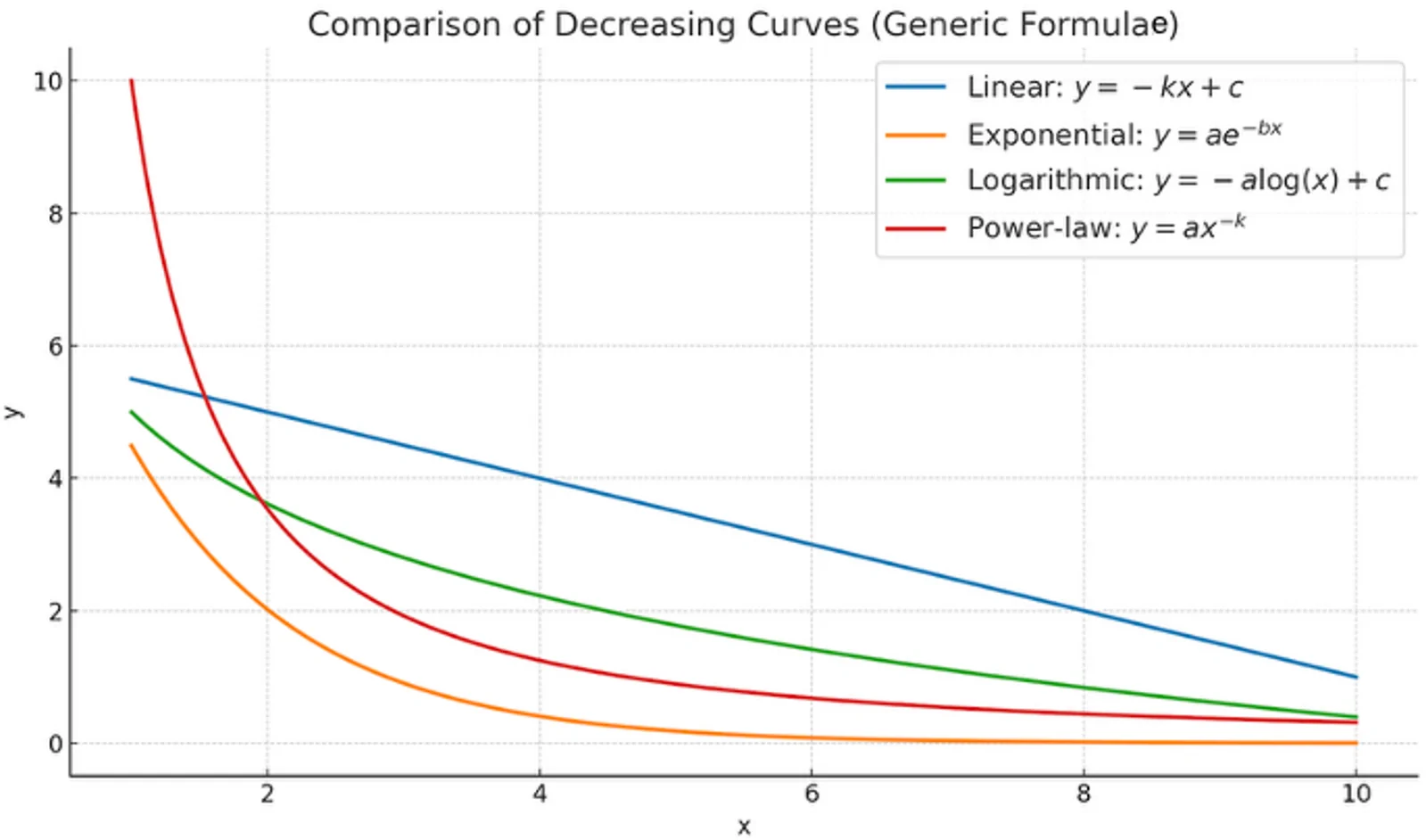
Comparison of Decreasing Curves, coefficients chosen to fit on the same plot

**Fig. 3:**
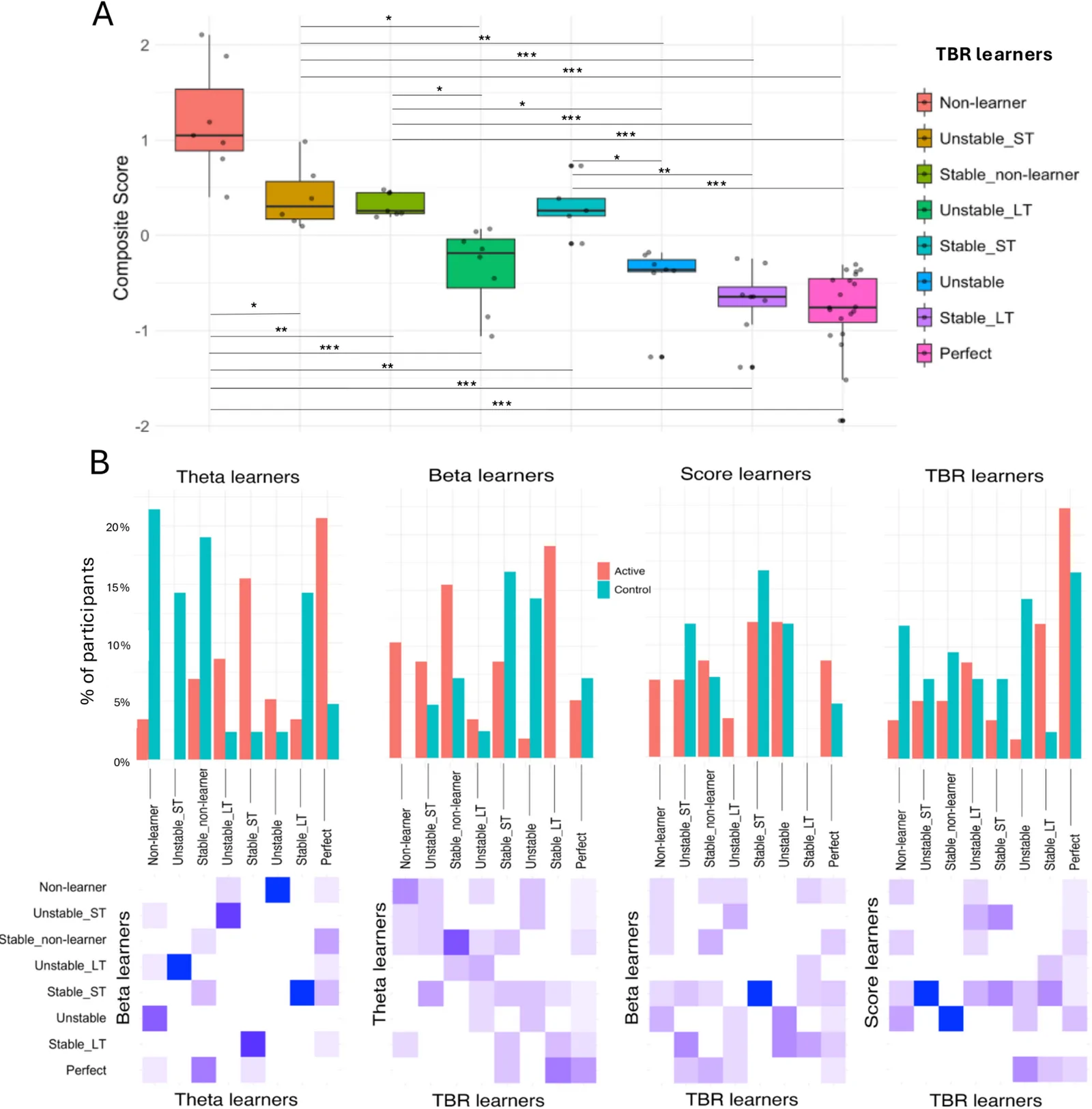
**A** Composite learning score (gain + focus + consistency) across eight TBR learner profiles reveal a three-tier structure. **B** Bar plots demonstrate distribution of participants across learner profiles. Control participants tend to cluster in Theta profiles associated with lower levels of learning, whereas Active participants are more frequently found in Beta profiles with similarly limited learning progress. A cross-tabulation matrix shows comparisons between different learner profiles across signal types, with each panel comparing two profile types. TBR-Theta diagonal dominance suggests some level of consistency. Theta-Beta off-diagonal cases demonstrate inconsistent learning across two signals

The first part of the results is devoted to descriptive and inferential analyses of learner profiles (Fig. 2). Specifically, group distributions (Active vs. Control) across learner profiles are summarized using frequency tables and visualized using bar plots (Table 1; Fig. 3B). To disentangle whether changes in TBR are primarily driven by theta or beta amplitudes, a cross-tabulation matrix was constructed (Fig. 3B). We also computed a composite score (gain + focus + consistency) and tested for differences among TBR learner profiles (Fig. 3A). By construction, lower (more negative) values reflect greater overall learning—decreasing TBR across sessions (gain), reduced variability (focus), and a more negative average within-session slope (consistency). One-way ANOVA and Tukey’s HSD post hoc tests were used for pairwise comparisons between specific learner types. Effect sizes and 95% confidence intervals were calculated.

RQ1 & RQ2 (theory-driven) – relationships between learning, non-specific effects, and symptom change.

Primarily, we needed to find the best model to explain variation in symptom change (RQ1) and learning profiles membership (RQ2) using a range of predictors. Thus, we built a forward stepwise predictive model by iteratively adding variables based on Akaike Information Criterion (AIC) statistic. Based on the predictive model, our statistical model constituted linear (RQ1) or logistic (RQ2) regression and analyzed its statistics.

*TBR*,* theta*,* beta* and *Score learner profiles* (with eight factors in each) served as training-specific independent variables (IVs) for RQ1 and DVs for RQ2. For all the models, non-specific predictors were *age (of the child at intake)*,* sex*,* race*,* head site* (Fz or Cz), *training site (OSU or NC)*,* hours of sleep*,* Neurofeedback Observation Rating System (NORS)*,* days from the beginning of treatment* and *medication status.* For each family of analyses, family-wise error rate (FWER) was controlled by *p*-value adjustment using the Holm-Bonferroni method.

**Fig. 4:**
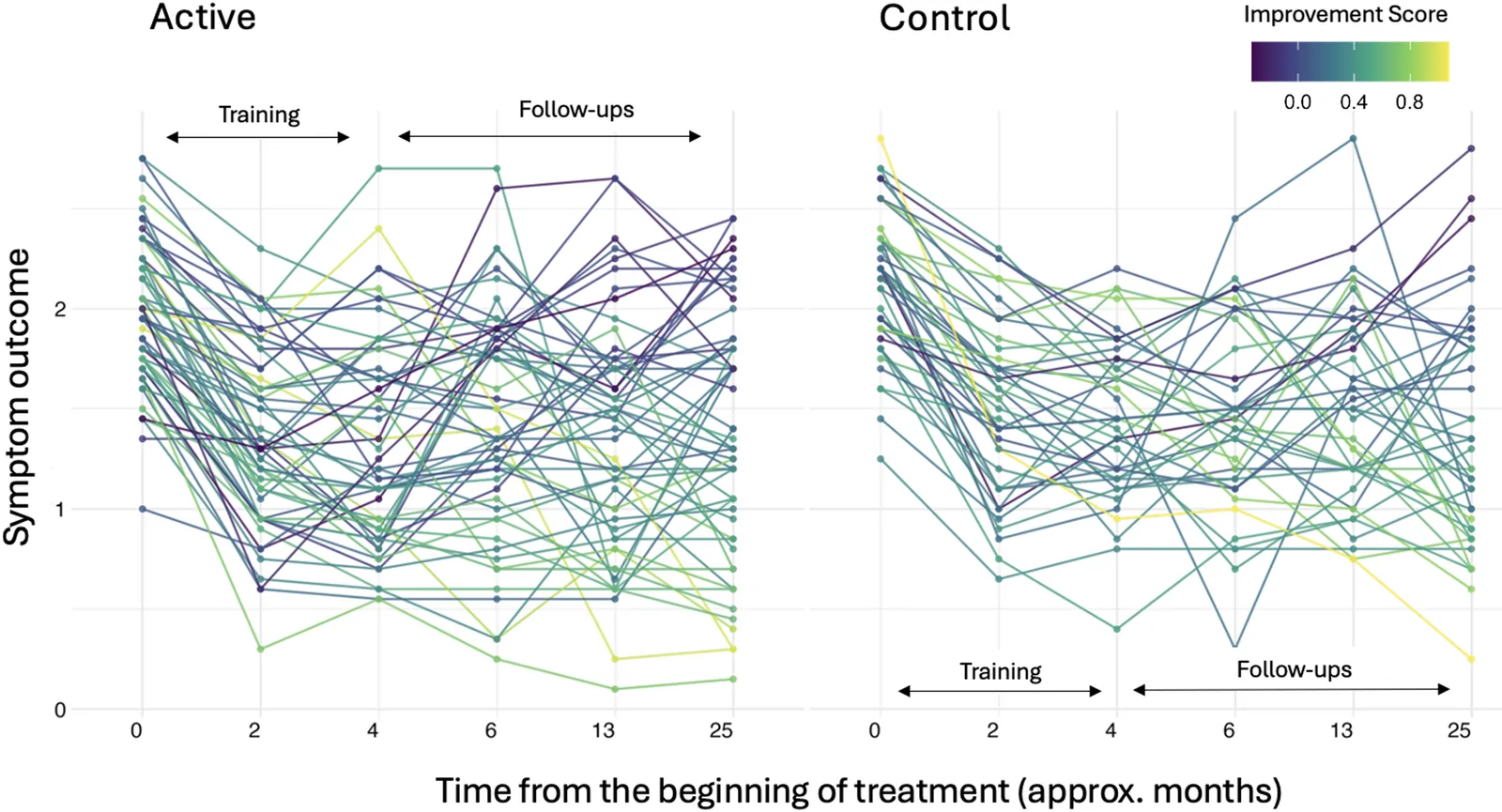
Each line represents an individual participant’s Conners DSM-5 ADHD Inattentive score over time. Color intensity reflects the magnitude of symptom improvement from baseline to end of treatment (including follow-ups). Facets separate participants by group. Symptom outcomes progressively decline across the training period, especially up to the first four months of training

To calculate the symptom change (served as DV in RQ1 models, Fig. 4) we modelled two metrics: *symptom gain training* that accounts for symptom change from pre- to post- treatment using linear regression model (difference between the ending point and the intercept), and *symptom gain long-term* which also accounts for 6-, 13-, and 25-month follow-ups.

NORS is a trainer-reported measure used to assess children’s participation during NFB sessions. It includes five parameters such as Cooperation, Understanding (of the task), Completion (ability to complete the entire session), Time Attending, and Remain Seated. To address potential rater bias in the NORS variable, which was scored by multiple trainers, we standardized NORS scores within each trainer. The z-scored NORS was then aggregated at the participant level and used as a predictor in the symptom outcome model.

As a separate question of interest, we investigated how five behavioral outcomes of NORS change across different blocks and built the linear mixed effect model with a random effect of participants to see if other non-specific factors predict NORS parameters.

RQ3. Does learning in neurofeedback follow distinct curve patterns?

To address RQ3 on the shape of the learning curve in NFB, we analyzed the shape of TBR amplitude change across sessions. This analysis was limited to Active participants classified as Perfect Learners in TBR amplitude, since they exhibited the ideal learning pattern, and learning in the Control group may be driven by different mechanisms.

To determine which model best fits the observed data, we compared different learning models: linear, exponential, power-law, and log-law (see Fig. 2 for model curves comparisons). In the first approach we fit separate models for each participant and calculated AIC per individual model, then counted how many participants were better fit by one model compared to another. In the second approach we fit one LMM across all participants, allowing random effects to account for individual differences. AIC values were then compared to assess overall model fit across participants.

### Data-driven Approach

**Fig. 5:**
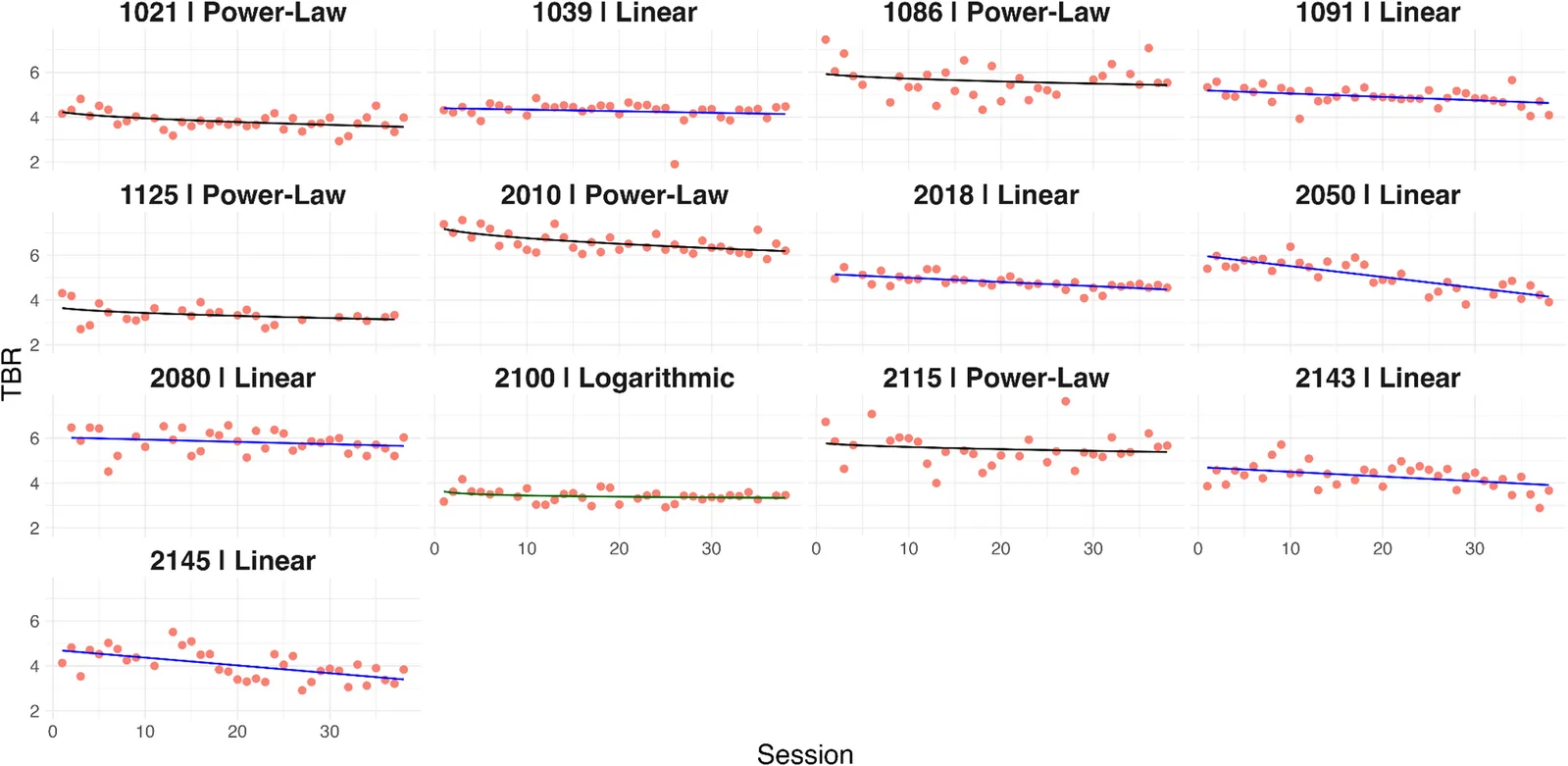
TBR learning trajectories of Active Perfect learners, curve family denoted by subtitle and color coding

**Fig. 6:**
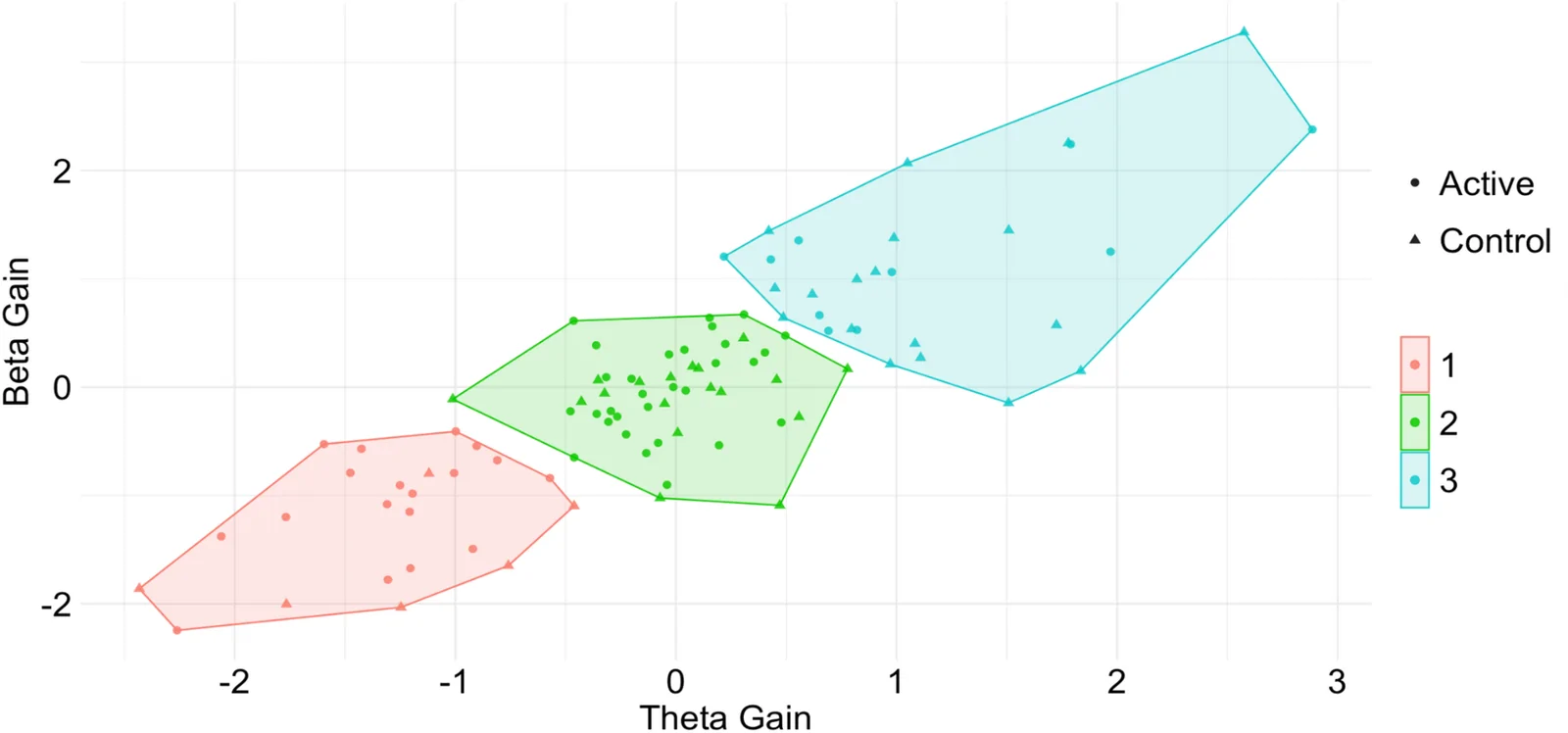
Theta-Beta clustering by group (Active vs. Control). Cluster 1 - successful in theta self-regulation, dominated by Active participants. Cluster 2 - no significant theta-beta change, possibly reflecting absence of learning across sessions. Cluster 3 - failure to decrease theta as intended, mainly consisting of Control participants

Although TBR was the primary outcome measure, it is a compound metric derived from theta and beta power (Fig. 5). Therefore, we analyzed theta and beta amplitudes separately to better understand their distinct contributions to the observed effects. A Pearson correlation was computed between the average theta and beta amplitudes across sessions to explore the relationship between these two modalities. A clear separation of participants into three general learning clusters (based on unsupervised *k*-means clustering analysis) was visualized in a cluster plot (Fig. 6).

RQ1 & RQ2 (data-driven) – relationships between learning, non-specific effects, and symptom change.

To address RQ1 & RQ2, we calculated our three metrics—gain, focus, and consistency—for all recorded training parameters, including those not directly trained: theta amplitude, theta percent-time-in-reward (under the threshold), beta amplitude, beta percent-time-in-reward (above the threshold), EMG amplitude, EMG percent-time-in-reward (below the threshold), Score and TBR.

For the following RQs 1 and 2 similarly to theory-driven analysis we used a forward stepwise predictive model by iteratively adding variables based on AIC statistical criteria. We then built linear (RQ1) or logistic (RQ2) regression and analyzed its statistics. For all the models, non-specific predictors were as in theory-driven approach, while training-specific factors vary depending on how we represented learning:

1. Learning as a set of all possible training parameters.

We first considered learning in its most granular form—by incorporating all individual learning parameters. *Theta amplitude gain*,* theta amp focus*,* theta amp consistency*,* theta time gain*,* theta time focus*,* theta time consistency*,* beta amp gain*,* beta amp focus*,* beta amp consistency*,* beta time gain*,* beta time focus*,* beta time consistency*,* electromyographic (EMG) amp gain*,* EMG amp focus*,* EMG amp consistency*,* EMG time gain*,* EMG time focus*,* EMG time consistency*,* Score gain*,* Score focus*,* Score within- session-slope*,* age*,* sex*,* race*,* head site* (Fz or Cz), *group*,* location of training*,* hours of sleep*,* NORS*,* days from the beginning of treatment* and *medication status* served as IVs and *symptom change* as DV.

2) Learning as cluster membership for each training parameter.

In this approach, we categorized participants based on their learning patterns for each training parameter. To achieve this, we applied the *k*-means clustering algorithm to three calculated metrics together (gain, focus, consistency) for each training parameter (e.g. TBR, theta amplitude, theta percent of time in the rewarding condition, etc.). To determine the optimal number of clusters, we primarily relied on the elbow method but incorporated other numeric or visual methods (e.g. gap statistic and Davies-Bouldin index). We assessed clustering quality using Silhouette plots. As a result, participants were assigned to one of the clusters (ranging from two to four per training parameter). This classification was performed without any predefined assumptions about cluster identities. We then built a stepwise regression with various clusters as IVs, iteratively adding variables based on statistical criteria, and used it to construct a linear model with *symptom change* as DV for statistical analysis.

3) Learning as membership in a general Cluster across all training parameters.

Finally, we explored learning as a composite construct, grouping participants into clusters that account for all training parameters simultaneously. In order to do so, we employed a cluster pattern profiling approach, where we integrated different cluster assignments from previous subchapter (e.g. *theta amplitude clusters*,* beta percent of time in the rewarding condition clusters*,* EMG amplitude clusters*, etc.), created a unique identifier that represents which clusters a participant belongs to across multiple parameters and summarized how many participants belong to each unique combination of cluster assignments. We then applied dendrogram visualization, silhouette analysis, and hierarchical clustering (cut-tree approach) to identify the natural structure of clusters and then reduce the number of clusters to a meaningful minimum, ensuring a balance between interpretability and data complexity. *Clusters (final reduced clusters)* and non-specific variables served as IVs and *symptoms change* as DV in the final linear regression model.

## Results

### Theory-driven

The results of clustering for TBR amplitude for 58 Active NFB participants and 42 Control participants assigned 68 participants into the various learning profiles (Active = 36, Control = 32). The distribution by group and learners’ profile can be seen in the table and plot below (Table 1; Fig. 3B), alongside the clusters for the trained parameters theta, beta, and Score, for comparison.

TBR *Perfect learners* (Active = 13, Control = 7) is the most populated profile for both Active and Control participants. The second-most populated profile is *Stable long-term learners* (Active = 7, Control = 1) for Active participants, and *Unstable learners* (Active = 1, Control = 6) for Control.

As shown in Fig. 3A, ANOVA test and post-hoc TukeyHSD pairwise comparisons did not reveal a significant difference in TBR composite score (including gain, focus and consistency) between *Perfect*, *Unstable long-term*, *Stable long-term*, and *Unstable learners*. In contrast, the *Non-learner* profile was statistically distinct from all other profiles (Fig. 3A). The remaining three profiles (*Stable non-learner*, *Stable short-term*, and *Unstable short-term*) also grouped together, demonstrating lack of statistical difference between each other. However, *Unstable long-term* and *Stable short-term* were not different between one another, suggesting some overlap between three-tier emerging structures.

**Table 1:** Distribution of participants across theta, beta, Score and TBR learner profiles. Note that participants with indeterminate responder status were excluded from profile assignment

Learner profile	Group	Theta learners	Beta learners	Score learners	TBR learners
Perfect learners	Active	12	3	5	13
	Control	2	3	2	7
Stable long-term learners	Active	2	11	0	7
	Control	6	0	0	1
Unstable learners	Active	3	1	7	1
	Control	1	6	5	6
Stable short-term learners	Active	9	5	7	2
	Control	1	7	7	3
Unstable long-term learners	Active	5	2	2	5
	Control	1	1	0	3
Stable non-learners	Active	4	9	5	3
	Control	8	3	3	4
Unstable short-term learners	Active	0	5	4	3
	Control	6	2	5	3
Non-learners	Active	2	6	4	2
	Control	9	0	0	5
**Total**	**Active**	**37**	**42**	**34**	**36**
	**Control**	**34**	**22**	**22**	**32**

The results of clustering for theta amplitude for 58 Active NFB participants and 42 Control participants assigned 71 participants into the learners’ profiles (Active = 37, Control = 34). *Perfect learners* (Active = 12, Control = 2) and *Stable short-term learners* (Active = 9, Control = 1) groups are the most populated among Active participants and Non-learners (Active = 2, Control = 9) and *Stable non-learners* (Active = 4, Control = 8) across Control participants. *Unstable learners* groups (both short-term and long term) have a smaller number of participants in general (4–6 per category).

The results of clustering for beta amplitude assigned 64 participants into beta learner profiles (Active = 42, Control = 22). Stable long-term learners (Active = 11, Control = 0) and Stable non-learners (Active = 9, Control = 3) are the most populated learner types for Active participants, and Stable short-term learners (Active = 5, Control = 7) and Unstable learners (Active = 1, Control = 6) for Control participants.

The results of clustering for Score assigned 56 participants into Score learner profiles (Active = 34, Control = 22; Table 1). Stable short-term learners (Active = 7, Control = 7) is the largest group for both Active and Control participants, together with Unstable learners (Active = 7, Control = 5) for Active participants.

In the TBR–Theta cross-tabulation matrix, the diagonal pattern suggests some level of consistency, indicating that participants especially classified as Perfect learners, Stable non-learners, or Non-learners in theta also tend to exhibit the similar learning profiles in TBR. Additionally, Stable short-term and Unstable learners in beta show a similar alignment with TBR profiles, while Theta-Beta off-diagonal cases demonstrate inconsistent learning across two signals overall.

RQ1 (theory-driven). How do learning and non-specific effects relate to symptom outcome?

The results of the forward stepwise linear regression model with *TBR*,* theta learner*, *beta learner*, and *Score learner* and non-specific variables as IVs indicate that NORS is the only one predicting the *symptom change training* based on AIC. A linear model showed a trend-level effect (β = -0.16, SE = 0.08, *t* = -1.96, *p* = 0.05, p-adj *ns*).

When the model was rerun with five components of NORS separately, *Remain Seated* and *Time Attending* emerged as the strongest predictors of symptom change following training. Better ability to remain seated was associated with greater symptom improvement (β = -0.54, SE = 0.16, t=-3.40, p.adj < 0.01), while increased time attending sessions negatively affected the symptom outcome (β = 0.41, SE = 0.18, t = 2.22, p.adj = 0.03, p-adj *ns*).

In turn, *Age (of a child at intake)* affected all five NORS parameters, most significantly *Remain Seated* and *Time Attending* scores: older participants remained seated longer (β = 0.21, SE = 0.05, t = 4.09, p.adj < 0.01) and attended to the task for a longer duration (β = 0.15, SE = 0.05, t = 3.23, p.adj < 0.05) than younger ones.

The omnibus ANOVA for aggregated average NORS across all components showed no significant effect of block. However, analyses of all individual NORS components revealed significant changes across blocks, particularly between Blocks 3–5 (Sessions 11–38) and Block 1 (Sessions 1–5). All components except Understanding showed a decline over time.

The results of the forward stepwise linear regression model with *symptom change long-term* as DV indicated that *age* is the only IV that improves the model. A linear model suggested a marginal effect of *age* (*b* = -0.04, *t* = -1.72, *p* = 0.09, p-adj *ns*).

Note that symptom outcomes progressively decline across the training period, especially up to the first four months of training (Fig. 4). During the follow-ups there is a noticeable rise in dispersion for both groups.

RQ2 (theory-driven). How is learning modulated by non-specific effects?

Stepwise multinomial logistic regression demonstrated that none of the non-specific predictors significantly improve the fit over base rates for the participants’ membership in specific TBR and theta learner profiles.

A multinomial logistic regression predicting beta learner profiles by non-specific effects revealed that *days from the beginning of treatment* (AIC = 400.98 vs. 402.91 in null model) is the only IV which would improve the model.

A multinomial logistic regression predicting Score learner profiles by non-specific effects revealed that *medication status* (AIC = 349.47 vs. 334.57 in null model) and *NORS* (AIC = 346.18 vs. 349.47 in medication status model) are IVs which would improve the model.

R3. Does learning in neurofeedback follow distinct curve patterns?

For Active participants from Perfect learners profile, model fitting results for TBR amplitude revealed that a linear model best described the change over the course of treatment in seven individuals (Fig. 5). Among these, strong support for the linear model (AIC difference > 2) was observed in four participants. A power-law model provided the best fit for five participants, while a logarithmic model was optimal for one (Fig. 6).

The second, group-level analysis indicated that the linear model provided a fit comparable to the logarithmic model and outperformed both the power and exponential alternatives (AIC_lin = 695.60, AIC_log = 696.12, AIC_power = 705.01, AIC_exp = 759.35).

### Data-driven

Exploring patterns of EEG self-regulation across participants we found a strong positive correlation (83%) between theta and beta amplitudes. An unsupervised clustering analysis based on individual learning gains in theta and beta amplitude demonstrated that participants can be effectively separated into three clusters: Cluster 1 (successful in theta self-regulation, dominated by Active participants), Cluster 2 (no significant theta-beta change, reflecting absence of learning across sessions), and Cluster 3 (failure to decrease theta as intended, mainly consisting of Control participants).

RQ1 (data-driven). How do learning and non-specific effects relate to symptom outcome?

1. Learning as a set of all possible training parameters.

The results of the most granular stepwise linear regression model indicated that *EMG focu*s (β = -0.10, SE = 0.05, t = -2.12, *p* < 0.05, p-adj ns), beta time focus (β = 0.14, SE = 0.05, t = 2.77, *p* < 0.01, p-adj ns), beta time gain (β = 0.08, se = 0.05, t = 1.77, p = 0.08, p-adj ns) and Score focus (β=-0.18, se = 0.05, t=-1.74, p = 0.09, p-adj ns) are training-related IVs that best predict the model with symptom change during training as DV based on AIC. Besides these, the results demonstrated that NORS (β = -0.18, SE = 0.09, t = -2.07, *p* < 0.05, p-adj ns), Sleep (β < 0.01, SE = 0.00, t = 1.77, *p* = 0.08, p-adj ns) and days from the beginning of treatment (β < 0.01, se = 0.00, t = 1.96, p = 0.05, p-adj ns) are non-specific IVs that improve the model. Note that, after applying FWER none of these effects remain significant.

The results of the forward stepwise linear regression model with non-specific effects and all individual learning parameters indicate that *age* (β= -0.04, SE = 0.03, t-value= -1.72, p-value = 0.09, p-adj *ns*) is the only IV that improves the model with *symptom change long-term* as DV.

2) Learning as cluster membership for each training parameter.

The results of the stepwise linear regression model with non-specific effects and clusters for each training parameter indicate that EMG amplitude clusters ( for the “no change” cluster in comparison with “non-learner”: β = 0.47, SE = 0.19, t = 2.43, *p* < 0.05, p-adj ns) and EMG time clusters ( for the “learner” cluster in comparison with “non-learner”: β = -0.32, SE = 0.17, t = -1.89, *p* = 0.06, p-adj ns) are training-related IVs that improve the model with symptom change during training as DV based on AIC. NORS (β = -0.21, SE = 0.09, t = -2.40, p < 0.05, p-adj ns) and days from the beginning of training (β = 0.00, SE = 0.00, t = 1.74, *p* = 0.08, p-adj ns) were non-specific effect which also improved the model.

3) Learning as membership in a general Cluster across all training parameters.

**Fig. 7:**
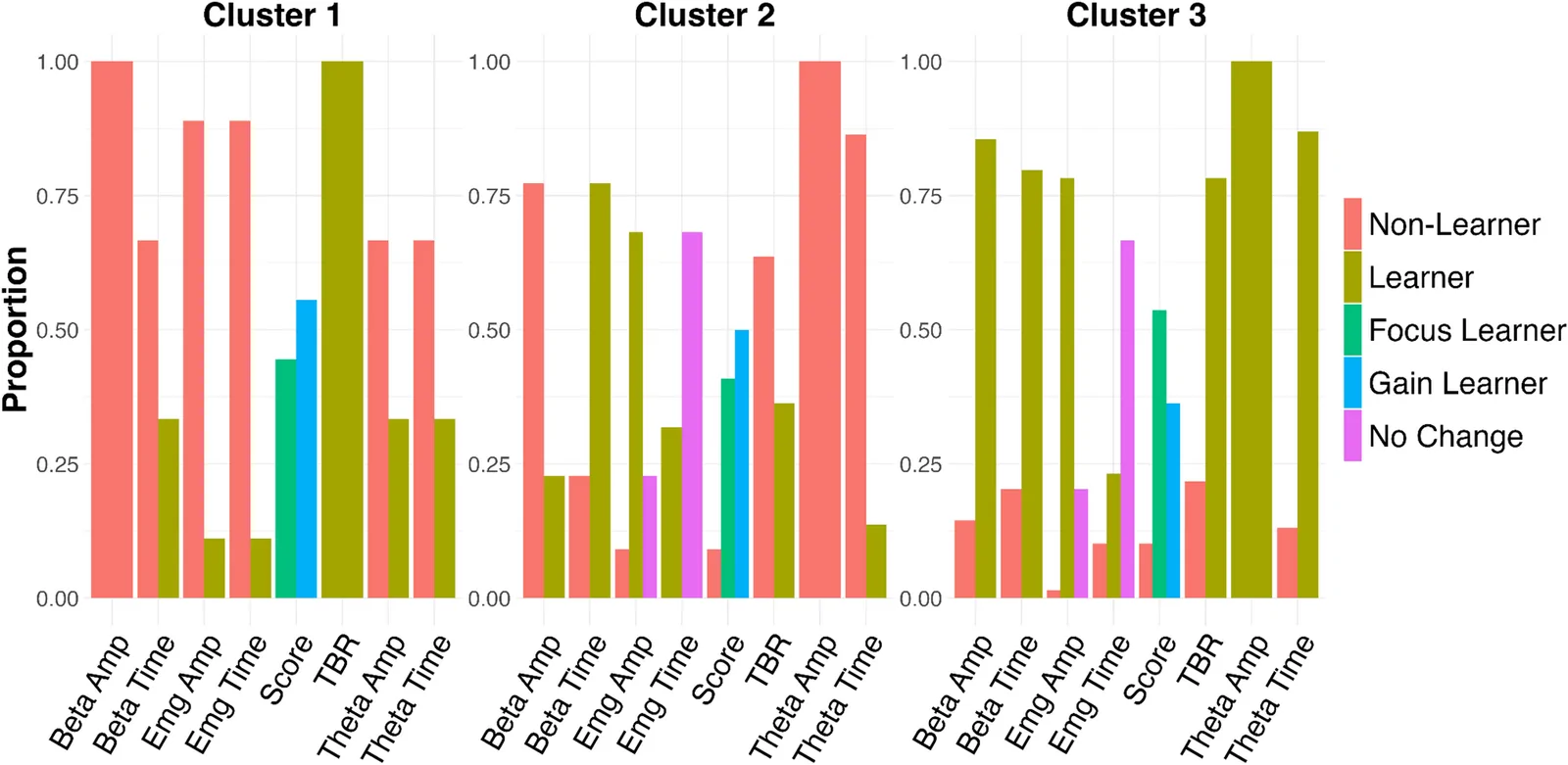
Distribution of learner profiles across General Clusters. Cluster 1 shows a predominance of beta non-learners and TBR learners. Cluster 2 is characterized by a high concentration of theta non-learners. Cluster 3 contains the majority of learners across parameters, reflecting broadly successful NFB engagement. Together, these patterns suggest distinct neurophysiological response profiles across clusters

Grouping participants based on all training parameters simultaneously, resulted in their classification into three distinct clusters — the optimal number determined by dendrogram analysis and supporting statistical criteria. Distribution of participants’ profiles in training clusters across general Clusters is illustrated below on Fig. 7.

The results of the stepwise linear regression model with non-specific effects and general Cluster across all training parameters indicate that only *NORS* (β = -0.16, SE = 0.09, t = -1.74, *p* = 0.08, p-adj *ns)* improves the model with *symptom change training* as DV, based on AIC.

R2 (data-driven). How is learning modulated by non-specific effects?

The investigation of whether non-specific variables affect the cluster classification for *TBR amplitude* did not reveal any results.

For *theta amplitude* results demonstrated that when not on ADHD medication the log-odds of being a learner is significantly higher than being a non-learner (β = 0.59, se = 0.09, t = 6.53, p.adj < 0.01) and are equal to 79.7%. When on ADHD medication the difference in probabilities is not statistically significant. Additionally, each additional minute of sleep was associated with a 1.3% decrease in the odds of being in the learner cluster *(*β *= -0.013*,* SE = 0.00; z=-25.44*,* p.adj < 0.001)*.

ADHD medication status also influences how participants are classified as learners or non-learners based on the percentage of time below the theta threshold (Residual Deviance = 119.73, AIC = 123.73). Similar to previous findings, participants who are not medicated are significantly more likely to be learners than non-learners (β = 0.49, SE = 0.10, t = 4.79, p.adj < 0.05), while for those on medication the difference between learners and non-learners disappears.

Multinomial logistic regression revealed that the *number of days from treatment onset* and *location of the training* were the best predictors for EMG time below the threshold learning profiles (Residual Deviance = 172.38, AIC = 184.38). Specifically, increased *days from treatment start* significantly increased the odds of being classified as a learner rather than a non-learner (β = 0.043, SE = 0.02, *p.adj* < 0.05) translating to about a 4.4% increase in the odds per day. OSU/NC *location of the training* parameter did not reach significance overall.

Forward selection identified *medication status* as the best predictor of general Cluster membership. When medication status was zero (participants were not medicated), strong and significant differences were observed between Cluster 1 (non-learners) and Cluster 3 (learners) (β = -0.69, SE = 0.07, t = -10.03, p.adj < 0.01), as well as between Cluster 2 (non-theta learners) and Cluster 3 (learners) (β = -0.58, SE = 0.09, t = -6.48, p.adj < 0.01). Among unmedicated participants, the probability of being assigned to Cluster 3 – which comprises the majority of learners across parameters – was highest (~ 76%), while participants on ADHD medication showed a lower likelihood of being assigned to this cluster (~ 50%). Nevertheless, the effect of medication on odds of being assigned to Cluster 3 (vs. Cluster 1) is not statistically significant at the conventional 0.05 level (β = -1.24, SE = 0.74, *z* = -1.68, *p* = 0.094).

## Discussion

This study advances the NFB field by offering both a theoretically grounded and a data-driven framework for characterizing learning as a dynamic, individualized process. This approach allows us to move past the question of whether NFB is effective and instead examine *how*, *for whom*, and *under what conditions* it yields the greatest therapeutic benefit. This is especially important given the field’s mixed and often inconclusive findings, which risk contributing to fragmented or inconsistent healthcare policies that directly affect patients.

In our study we used a multi-component parameterization of learning to observe that NFB performance is not a major determinant of symptom improvement. Instead, contextual and interpersonal factors—such as participant age, trainer experience, and engagement in task—appeared to play a more significant role in determining treatment outcomes. Additionally, our analysis highlights that learning curves in NFB do not follow a uniform shape. These varying curve profiles may reflect distinct underlying learning mechanisms, aligning with either operant-conditioning models or skill-acquisition frameworks, depending on individual learning trajectories.

In the following discussion, we begin by exploring the insights derived from the learner profiles and then address our three main research questions.

### Analyses of Learner Profiles

Although not a separate research question, our learner-profile analysis verified target engagement and characterized heterogeneity in NFB response. Profile membership—derived from gain, focus, and consistency—shows whether and how participants acquired self-regulation, and the emerging structure offers a practical basis for tailoring protocols.

The most populated TBR learners profile is Perfect Learners, representing the ideal NFB outcome. Belonging to this group suggests strong self-regulation, learning retention, and engagement. The number of participants classified as Perfect Learners in TBR exceeds that of any other training parameter: theta, beta, or Score (Table 1). This suggests TBR is particularly responsive to NFB and may reflect a more trainable or plastic neural marker. This effect is especially pronounced in the Active group, which contains almost two times as many TBR Perfect Learners as the Control group. Conversely, Control participants are more than twice as likely to show no TBR regulation improvements (non-learner profile) and are more likely to be in groups with less learning implied, confirming that sham feedback does not facilitate learning, while real NFB worked as intended for TBR self-regulation.

Nevertheless, there are several Control participants in high-performing TBR profiles, such as Perfect or Unstable learners. It is possible that Control participants exhibited genuine improvement due to an inverse mechanism—where enhanced cognitive focus or sustained attention during the task led to favorable shifts in brain oscillations, rather than the feedback causing those shifts to thus promote focus and attention. This could have led to subtle changes in brain activity—slight decreases in theta and increases in beta—that were not sufficient to classify them as learners in either domain individually but together reduced the TBR ratio. Second, it is possible that, by chance, the sham signal occasionally resembled their actual brain activity, inadvertently producing effects similar to those seen in the Active group. However, this was controlled in the study and did not exceed 7%.

Interestingly, the presence of Control participants in TBR Perfect Learners profile was not supported by corresponding membership in the TBR’s component metrics, theta and beta. In fact, Control participants are largely absent from Perfect learners profile in both theta and beta clustering. The fact that TBR does not directly reflect the systematic regulation of theta and beta was also supported by other results, showing that, for example, the Stable short-term learners group is relatively well populated for both theta and beta individually, but barely present in TBR.

The second most populated group for TBR amplitude is Stable long-term learners. These individuals do not exhibit consistent improvements within sessions but show steady, gradual gains across sessions, presumably effectively engaging longer-term consolidation mechanisms. Importantly, the TBR Perfect learner profile’s composite score is not different from Stable long-term learners (see Fig. 3A), placing them close to Perfect Learners in overall learning quality.

Analysis of differences in composite score between eight learner profiles revealed a higher-order structure, possible grouping into three broader functional categories. The high-learning profiles (Perfect, Unstable long-term, Stable long-term and Unstable learners) clustered together, showing no significant differences from one another—likely underscoring long-term gain as the most robust indicator of learning progress. Non-learner profile was statistically distinct from all other profiles, suggesting a clear lack of desired NFB response and pointing to the potential need for alternative interventions. This reliable separation supports the validity of the metrics—gain, focus, and consistency—in capturing meaningful learning patterns, particularly at the extremes. The remaining three profiles (Stable non-learner, Stable short-term and Unstable short-term learners) also grouped together, forming a third, intermediate category characterized by partial or inconsistent learning. This emerging three-tier model may serve as a basis for tailored support strategies and protocol adjustments. Nevertheless, these results should be viewed cautiously due to subgroup overlap and a constrained sample.

Taken together, our findings indicate that TBR is the most responsive target for training, with Active participants over-represented among high-learning profiles and Control participants concentrated among non-learners. The emergent three-tier structure (high, intermediate, non-learner) appears psychometrically meaningful—clearly separating robust acquisition from absence of learning—yet it does not predict symptoms. Its value is therefore operational: stratify analyses and randomization by tier and apply early decision rules – probe transfer in the high tier, adapt/scaffold in the intermediate tier, and pivot early for non-learners. In the next section, we discuss what predicts downstream clinical improvement.

RQ1. How do learning and non-specific effects relate to symptom outcome?

In this study we examined whether indices of NFB learning—or non-specific engagement factors—predict symptom change, directly targeting a gap highlighted in prior work, which has questioned if and when learning contributes to efficacy (Kuznetsova et al., 2023; Zuberer et al., 2015). When predicting symptom improvement by parameters of learning, none of the effects from regression models survived correction for multiple comparisons. The coefficient estimates of predictors that were significant before FWER represent *small effects* in practical terms, casting doubt on their robustness. The direction of some effects (e.g., negative for EMG and Score focus, positive for beta time gain) misaligned with theoretical expectations, which adds doubts to the face validity. Thus, we can claim that none of the learning parameters appeared to be predicting success with symptom outcome. This result conforms with previous literature, which suggested that sometimes learning itself is not required for positive outcomes (Janssen et al., 2017; Veilahti et al., 2021). For example, in Janssen et al. (2017), individual learning curves were not significantly correlated with behavioral change. Although children with ADHD learned to modulate EEG states during NFB, the absence of behavioral correlates suggested limited transfer to everyday functioning. In similar fashion, Veilahti et al. (2021) showed behavioral scores from the Test Of Variables of Attention, and symptoms from adult ADHD Self-Report Scale, appeared not to be directly influenced by NFB learning; they were instead mediated by scores for dissociative experiences and the behavioral inhibition system. Furthermore, this pattern is consistent with the possibility that in-session self-regulation reflects acquisition under training conditions, whereas clinical benefit may depend more strongly on transfer—the ability to apply the learned regulation strategies in daily contexts when symptoms are elicited (Hasslinger et al., 2022). This distinction parallels motor-learning models in which acquisition is necessary but not sufficient, and transfer/generalization determines functional outcomes (see, e.g., Kantak & Winstein, 2012).

Our results suggest that non-specific engagement in training (e.g. NORS) may play a role in influencing symptom changes during treatment. Specifically, training engagement can positively affect the reduction of symptoms. Upon breaking down NORS into its components, we found that a better ability to remain seated was linked to greater symptom improvement, while, counterintuitively, increased time spent attending sessions was associated with symptom deterioration. However, after adjusting NORS scores for trainer-related scoring biases, TimeAttending was no longer a statistically significant predictor of symptom improvement. This suggests that the initial association may have been driven, at least in part, by trainer-specific scoring tendencies rather than a true effect of time spent attending the task. Note that older participants on average remained seated longer than younger ones and attended to the task longer, which is in line with background literature (Biederman et al., 2000; Kessler et al., 2005; Shaw et al., 2007).

The finding that *RemainSeated* emerged as the strongest predictor of symptom improvement aligns with results from our data-driven analysis, in which muscle activity (EMG) also surfaced as a factor associated with symptom change across sessions—albeit sometimes in unexpected directions. This connection is intuitive, as the ability to remain seated likely reflects a participant’s ability to regulate muscle activity, suggesting a plausible link between behavioral engagement and physiological control.

All NORS components except Understanding showed a decline over time, suggesting that while children’s comprehension of the task improved, other aspects of participation—such as Cooperation, Completion, Time Attending, and Remain Seated—decreased as the sessions progressed, possibly reflecting task demand, which increased with trial length. Given the observed association between remaining seated and symptom improvement, session duration represents a promising target for future investigation in both research and protocol design.

It is also reasonable that training engagement predicted symptom improvement during the training period but not when follow-up sessions are taken into account, as it likely reflects only immediate session participation. When follow-up outcomes were included in the model, age (of the child at the time they entered the study) emerged as a significant predictor, suggesting that it may have a small but positive influence on longer-term symptom change. Several factors may account for this association. First, younger children may naturally outgrow certain symptoms, such as hyperactivity and impulsivity, as part of typical developmental trajectories (Biederman et al., 2000; Shaw et al., 2007). Second, older participants may acquire compensatory cognitive or behavioral strategies over time, enabling them to better manage their symptoms, independently of short-term interventions (Kessler et al., 2005). In sum, neural learning did not translate into reliable symptom gains; the only modest effects on symptoms were tied to general engagement (especially remaining seated) and to age in the model with follow-up assessments up to 25 months.

The difficulty of achieving consistent efficacy and specificity in NFB training for ADHD may stem from the nature of the target process itself. NFB approaches with high specificity—such as fMRI-based protocols—tend to show stronger effects, as participants can achieve concrete results quickly through well-controlled intra-session learning and clearly defined outcomes (Birbaumer et al., 2009; Leibovitz et al., 2022). Similarly, when NFB is used in domains like sports, participants often know exactly what to focus on: they can imagine and feel the desired state in their body, which makes the training goal both tangible and actionable. By contrast, “attention” without context is an abstract construct—what exactly does it mean to attend, and how should it feel, when no target of attention is defined? The lack of specificity in the training target may explain why NFB for ADHD has shown limited and inconsistent effects on symptom outcomes (Cortese et al., 2016; Neurofeedback Collaborative Group, 2021; Peterson et al., 2024). Protocols that ask children to self-regulate without providing clear, embodied anchors may not align with the nature of the learning process. Instead, interventions may be more effective when linked to concrete, bodily states—such as sitting still or sustaining gaze—outcomes that are both perceivable by the participant and measurable, as also reflected in our results. Taken together, these findings caution against equating successful EEG regulation with clinical benefit and argue for designs that cultivate and measure real-world transfer, minimize rater bias, and tailor protocols to developmental stage. In the next section we discuss how and if NFB learning is modulated by non-specific effects.

RQ2. How is learning modulated by non-specific effects?

Understanding what predicts learning in NFB is pivotal because learning is the proximal engine the intervention is designed to train, yet there is wide variation in who does learn. Without explaining that variability, we cannot know whether inconsistent efficacy reflects mismatched conditions rather than an absent mechanism.

Thus, we examined whether non-specific factors predict learner status across training targets. Several models revealed a consistent trend: participants not on ADHD medication had significantly higher odds of being classified as learners compared to non-learners, while taking medication seems to flatten or blur that distinction — making it harder to differentiate between learners and non-learners. Thus, medication might reduce natural learning differences (normalize performance) and the variation between learners and non-learners.

Sleep had a counterintuitive effect in our model, suggesting that each additional minute of sleep slightly decreases the odds of being in the theta learner cluster. Longer sleep may lower daytime sleep pressure (drowsiness) and thus reduce baseline theta, leaving less “headroom” to down-train. With less excess theta to suppress, observed reductions are smaller, making theta-learner classification less likely (a floor effect).

Our results also suggest that a longer treatment duration — measured as the number of days elapsed from the start to the end of training — was associated with better EMG control (higher likelihood of being classified as EMG ‘learner’). It may reflect a spacing or consolidation effect, whereby having more time between sessions facilitates better behavioral adaptation, including improved muscle control. 

There were no non-specific factors affecting membership in TBR or beta learning profiles. TBR learning appears less sensitive to common non-specific factors than theta or EMG. This aligns with our broader pattern in which TBR is the most trainable target and most clearly separates Active from Control conditions, suggesting that feedback contingency dominates the variance and leaves little room for moderators to emerge. For other targets, future interventions should account for medication status in stratification and analysis and interpret learning estimates cautiously when participants are medicated, as learnability differences may be masked. Programs should monitor sleep and adjust scheduling or use brief arousal calibration to avoid very low sleep-pressure states when training theta, and leverage spacing where feasible, as longer elapsed intervals may support EMG control. In the next section we discuss whether the learning process itself follows distinct curve families and what underlying mechanism they can reveal.

RQ3. Does learning in neurofeedback follow distinct curve patterns?

Learning curves are a window into how NFB works—not just whether learning occurs. From stage-based accounts of skill acquisition (Fitts & Posner, 1967; Karni & Sagi, 1993; Kuznetsova et al., 2025), we would expect trajectories to be non-uniform and multi-phasic. To test this, we modeled individual TBR session-by-session trajectories with competing functional forms (linear, power, logarithmic, exponential) and evaluated evidence at both participant and group levels. Our aim was to determine whether a single canonical curve function suffices to map curve shapes onto plausible mechanisms (reinforcement-like vs. skill-acquisition dynamics).

Our results favored a linear trajectory for most participants. This converges with Janssen et al. (2017), where introducing a quadratic term did not yield a better fit than a linear mixed model children’s theta and beta activity over the treatment period. Nevertheless, in our study only a few participants showed strong evidence for any single model (four strongly favored linear), underscoring that NFB learning does not follow a single, consistent pattern. Many learners showed non-monotonic patterns that do not conform to smooth, continuous functions of model curves (Fig. 5). Note also that AIC used to assess the best fit penalizes model complexity: if the data don’t strongly support the additional complexity, AIC will favor the simpler linear model that might explain its dominance in our results (Sutherland et al., 2023).

The observation that some participants exhibit steady, linear improvement while others show non-linear trajectories—such as rapid early gains that slow over time—offers valuable insight into the underlying mechanisms of NFB learning. However, these learning trajectories did not reliably predict ADHD symptom improvement, which limits how strongly such decision rules can be justified clinically at this stage.

The skill acquisition model of learning can manifest as non-linear or discontinuous shifts, for example when a learner aggregates knowledge and abilities into a higher-order representation via ‘chunking’. Empirical NFB studies report that learners often go through exploratory phases of mental strategy testing, followed by abrupt gains in control once a successful approach is discovered (Gaume et al., 2016; Davelaar, 2018). Some authors describe these transitions as phase shifts—a brain–behavior switch into a new, more stable regulatory state (e.g., Stoller, 2011)—though this remains speculative without direct neurophysiological corroboration.

In some cases, learners may exhibit rapid early progress not solely due to strategy discovery, but because of latent capacities — pre-existing but previously unexpressed abilities to acquire specific skills. These may include intuitive pattern recognition, heightened interoception, or cognitive flexibility that facilitates faster adaptation to training demands (Van Der Veer et al., 2020). Together, these frameworks suggest that the most successful learners may be those who combine exploratory strategy use with internal model updating—resulting in non-linear learning curves with steep early gains followed by stabilization.

In contrast, operant conditioning, based on incremental reinforcement, in principle yields more gradual, continuous changes in behavior or EEG patterns. This associative learning process emphasizes trial-and-error adaptation, where desired EEG features become more frequent over time through repeated reward without substantial strategic engagement (Lubianiker et al., 2022; Staddon & Cerutti, 2003).

Differences between models may give rise to distinct learning dynamics during NFB. For instance, when feedback is withdrawn, learners relying on skill acquisition may retain their performance due to internalized self-regulatory strategies, whereas operant learners may show a sharp decline, indicating reliance on external reinforcement. Similarly, when task parameters change unexpectedly, strategic learners may adapt more easily through cognitive flexibility, while associative learners may struggle, or require re-conditioning. Interestingly, the structure of the training—in our case where each block increases in duration by one minute every five or ten sessions—may also interact with these mechanisms. Longer sessions could amplify fatigue effects in later stages of training, disproportionately affecting strategy-driven participants, while associative-learning participants might continue to improve steadily despite the added difficulty, potentially because their performance is more reliant on habituated response patterns. Future research should explore how protocol structure interacts with individual learning styles, potentially tailoring session design to learner profiles to optimize outcomes.

Together, these findings support a heterogeneous view of learning in NFB, in which the nature of the learning process (strategic vs. associative, operant-conditioning vs. skill-acquisition, etc.), the changing task demands (e.g., session length) and the basal state of the subject may contribute to the diverse trajectories observed across individuals.

In sum, this work contributes a theory-informed, data-driven account of NFB learning that (i) introduces a multi-component learner-profiling framework (gain, focus, consistency), (ii) identifies TBR as the most trainable target with Active participants concentrated among high-learning profiles, and (iii) demonstrates a dissociation between successful EEG regulation and clinical improvement, with symptom change tied more to non-specific engagement and developmental factors than to canonical learning metrics. Our results not only conform to but substantially extend the evidence base, addressing the sample size and methodological limitations of earlier studies.

Taken together, these patterns translate into concrete clinical actions. Practitioners should individualize protocols by tier: probe transfer and taper reinforcement in the high-learner tier; scaffold strategy use and strengthen long-term consolidation for intermediate tier; and pivot early to adjunct or alternative treatments for non-learners. Engagement should be designed, quantified, and de-biased (e.g., standardized NORS scoring, EMG, sleep, task length monitoring), with expectations and support tailored by age. Interventions should, where feasible, stratify or adjust for medication status and may benefit from linking targets to concrete, embodied states—such as remaining seated or sustaining gaze—that are both perceivable to the participant and objectively measurable. Together, these steps shift the emphasis from ‘does NFB work?’ towards ‘for whom, by which mechanisms, and under what conditions does it yield meaningful, durable benefit?’.

### Limitations

Interpretation of our results is tempered by constraints on how learning was classified, what was measured, and the granularity of those measurements. Below we detail these issues and outline refinements.

We defined eight learner profiles based on three dichotomized metrics: gain, focus, and consistency. While our overall sample was large for the field, splitting it across eight profiles led to small subgroups, raising the risk of artificial distinctions. As such, the classification may over-specify learning differences beyond what the data can reliably support — a point underscored by the lack of significant differences between some TBR learner profiles in our study. By refining the profiles—such as by combining them into broader, data-driven categories—we may reveal more robust learning dimensions beyond the current eight-profile framework. While a preliminary pattern of three-tier model in our data appears promising for early identification and individualized support, these findings should be interpreted cautiously due to subgroup overlap and sample constraints.

Our results demonstrate strong positive correlation between theta and beta amplitudes. This fact indicates that participants may not be learning to independently control theta and beta in opposite directions. Thus, the protocol design might need stronger differential reinforcement to separate the desired effects. Otherwise, it might have evoked undesired strategies by participants, e.g. self-limiting behaviors.

A related limitation lies in our focus on the TBR, which—despite its widespread clinical use and apparent responsiveness to training in our study —presents interpretive challenges. As a composite ratio, TBR conflates changes in two physiologically distinct processes: theta (often linked to drowsiness, mind-wandering, or control effort) and beta (often linked to alertness, sensorimotor tone, or EMG contamination) (Borrani et al., 2022; Cavanagh et al., 2009; Engel et al., 2001). A shift in the ratio can arise from decreased theta, increased beta, or both, and these routes need not reflect the same mechanism of regulation. Moreover, improvements in TBR do not guarantee component-level coordination: our data indicate that learners can show ratio changes without consistent, directional modulation of theta and beta individually, complicating the interpretation of TBR shifts.

Furtheron, our analysis relied on point estimates of TBR derived from task-level aggregated raw EEG values, rather than continuous measures. This method may overlook meaningful fluctuations within tasks, potentially missing brief regulatory successes/failures, and phase-like transitions. As such, future research should incorporate more granular, time-resolved EEG analyses to better characterize individual learning trajectories.

Lastly, we found limited evidence that NFB learning predicts symptom improvement. In-session EEG modulation may primarily reflect acquisition during training, whereas clinical benefit may depend on transfer to everyday contexts (Hasslinger et al., 2022). However, transfer was not directly assessed in the present study (e.g., no no-feedback transfer trials, ecological measures of strategy deployment, or ambulatory assessments). Future work should incorporate explicit transfer probes and real-world assessments to determine whether clinical improvement depends on deploying regulation during everyday attentional challenges rather than solely demonstrating control during training.

## Conclusion

This study set out to investigate the nature of learning in NFB, how it unfolds over time, and whether it meaningfully contributes to symptom improvement in ADHD. By moving beyond simplified classifications and average trends, we reveal the nuanced and heterogeneous nature of NFB learning. While TBR emerged as particularly responsive to training, individual learning trajectories did not reliably predict clinical outcomes. Instead, contextual and interpersonal factors, such as age, trainer experience and engagement in training, may play a more central role in treatment outcomes. We also demonstrate that the shape of learning curves—often overlooked in standard NFB studies— do not conform to a single shape; and can reveal underlying learning mechanisms that may align with either operant-conditioning or skill-acquisition type of learning. These findings offer a foundation for more individualized approaches to NFB training, with learner profile trends providing a basis for tailored support strategies and protocol adjustments.

## Data Availability

https://www.openicpsr.org/openicpsr/project/198003/version/V1/view%3Bjsessionid%3D5FE9F403AE469B447CF33C73A69C1A61?path=%2Fopenicpsr%2F198003%2Ffcr%3Aversions%2FV1%2FData_Files. Under this link one can access CSV/Excel data files plus study metadata and notes about design, measures, and follow-ups.
